# The neural correlates of the unified percept of alcohol-related craving: a fMRI and EEG study

**DOI:** 10.1038/s41598-017-18471-y

**Published:** 2018-01-17

**Authors:** Yuefeng Huang, Anusha Mohan, Dirk De Ridder, Stefan Sunaert, Sven Vanneste

**Affiliations:** 1 Lab for Clinical & Integrative Neuroscience, School of Behavioral and Brain Sciences, The University of Texas at, Dallas, USA; 2 Department of Surgical Sciences, Section of Neurosurgery, Dunedin School of Medicine, University of Otago, Dunedin, New Zealand; 3 Translational MRI, Department of Imaging and Pathology & Medical Imaging Research Center, Department of Radiology, Katholieke Universiteit Leuven - University of Leuven, Leuven, Belgium

## Abstract

Alcohol addiction is accompanied by aberrant neural activity. Previously, task-based fMRI and resting-state EEG studies have revealed that craving, a critical component of addiction, is linked to abnormal activity in cortical regions including the dorsal anterior cingulate cortex (dACC), nucleus accumbens (NAcc), posterior cingulate cortex (PCC) and pregenual anterior cingulate cortex (pgACC), etc. In this study, we combine these two imaging techniques to investigate a group of alcohol-addicted patients and provide convergent evidence for the neural correlates of craving not only in alcohol but substance abuse in general. We observe abnormal BOLD signal levels in the dACC, NAcc, pgACC, PCC, amygdala, and parahippocampus (PHC) in a cue-reactivity fMRI experiment. These findings are consistent with increased beta-band activity in the dACC and pgACC in resting-state EEG. We further observe desynchronization characterized by decreased functional connectivity in cue-based fMRI and hypersynchronization characterized by increased functional connectivity between these regions in the theta frequency band. The results of our study show a consistent pattern of alcohol craving elicited by external cues and internal desires. Given the advantage of superior spatial and temporal resolution, we hypothesize a “central craving network” that integrates the different aspects of alcohol addiction into a unified percept.

## Introduction

Alcohol consumption is a global phenomenon characterized by its pleasurable effects in social and personal settings. Physiologically, alcohol consumption is guided by the motivational incentive of the substance controlled by the mesolimbic pathway involving mainly the nucleus accumbens (NAcc) and the ventral tegmental area (VTA)^1^. People will generally drink alcohol for two reasons: (a) to feel better (reward drinking), or (b) not to feel bad (relief drinking)^2–4^. It is therefore not surprising that alcohol dependence is associated with one or more simultaneously present co-morbidities, especially mood disorders (27.6%), anxiety disorders (23.5%), and personality disorders (39.5%)^5,6^. In reward-drinking, an increase in the consumption of alcohol increases the release of dopamine and endogenous opioids in the brain, creating a feeling of reward^1^. However, excessive consumption of alcohol leads to alcohol dependence, which is characterized by impaired control over drinking, compulsive drinking, preoccupation with drinking, etc.^7^. Development of alcohol dependence involves a series of behavioral and neurophysiological events such as increased tolerance to alcohol itself, which leads to (1) increased consumption to obtain the same pleasure; (2) a clear dissociation between “liking” the pleasure and “wanting” the substance itself leading to a pathological craving of alcohol; and (3) development of other symptoms such as stress, anxiety, depression, restlessness, fever, headaches, etc., even within 24–48 hours of not consuming alcohol^1,8^. Thus, relief-drinking involves consuming alcohol to find a relief for these symptoms, forcing the patient to relapse into alcohol consumption^9,10^.

From a systems perspective, it is hypothesized that the motivational incentive creates a salience, characterized by increased arousal and attention to the stimulus, in this case alcohol^8^. This may be compensated for by consumption of more alcohol to achieve the same pleasure. However, in substance addiction, salience may lead to a change in the internal state of the subject to a new pathological “addicted” state^11^. This pathological state is proposed to be one in which the reward system is suppressed and is hence incompetent to compensate for the salience, thus triggering pathological craving^12^. This shift in self-referential state is called allostasis and may reflect the brain maladaptively compensating for the dysfunctional reward system^11,13^.

With the advent of noninvasive brain imaging techniques such as functional magnetic resonance imaging (fMRI) and electroencephalography (EEG), we can objectively determine the neural correlates of alcohol craving, highlighting the effects of reward and relief drinking in the brain. This may be achieved by investigating the resting-state and cue-reactive response of the brain to images of alcoholic and non-alcoholic beverages in patients with alcohol dependency using resting-state EEG and task-based fMRI. Previous studies have shown increased BOLD signal in response to alcohol-related cues in regions of the mesolimbic reward system such as NAcc, the ventral striatum (VS), and the amygdala (AMG)^14,15^. This was also accompanied by an increase in BOLD signal in other cortical regions, such as the dorsal anterior cingulate cortex (dACC), pregenual anterior cingulate cortex (pgACC), posterior cingulate cortex (PCC), orbital frontal cortex (OFC), precuneus, insula, and parahippocampus (PHC)^16–18^. These findings were consistent with studies reporting abnormal resting-state brain activity in alcohol-addicted patients after a period of abstinence. Several resting-state EEG studies report craving in alcohol addicts is related to increased spontaneous brain activity in the beta frequency band^7,19–21^, and relapse is associated with recurrence of gamma-band activity in dACC and PCC in alcohol addicted patients compared to healthy people^7^.

Although cue-reactivity and resting-state neurophysiological data independently provide evidence for changes in cortical and subcortical activity in alcohol addicts, to our knowledge there is no study that integrates both techniques in the same study. fMRI and EEG present their advantages and limitations in terms of spatial and temporal resolution. The high spatial resolution of fMRI aids in investigating activity of subcortical structures whereas the superior temporal resolution of EEG aids at looking at activity of cortical structures in different oscillatory bands. Changes in brain activity with a cue-reactivity paradigm represent the correlates of pathological craving in response to alcohol cues. Changes in spontaneous brain activity represent the same in the resting state. Thus, by combining the two we may be able to confirm the correlates of pathological craving, supporting a possible change in the state of the brain (i.e. allostasis) with high spatial and temporal resolution. Furthermore, compared to changes in BOLD signal and neural activity in the regions described above, changes in functional connectivity between these regions have been less explored both using fMRI-based cue-reactivity paradigms and resting state EEG.

Therefore, in the current study, we perform both cue-reactivity functional magnetic resonance imaging (fMRI) and resting-state electroencephalography (EEG) on a group of 11 alcohol-addicted patients 24 hours following alcohol abstinence to investigate the neural correlates of alcohol-related craving. We hypothesize changes in BOLD signal, resting state EEG activity, and functional connectivity, in cortical and subcortical regions reported in previous research confirming the neural correlates of craving. The observed changes will demonstrate characteristics of reward and relief drinking. We therefore propose a “central craving network” that may encode craving in not just alcohol addiction but in substance abuse in general.

## Results

### Cue-reactivity fMRI BOLD signal activation

**Figure 1:**
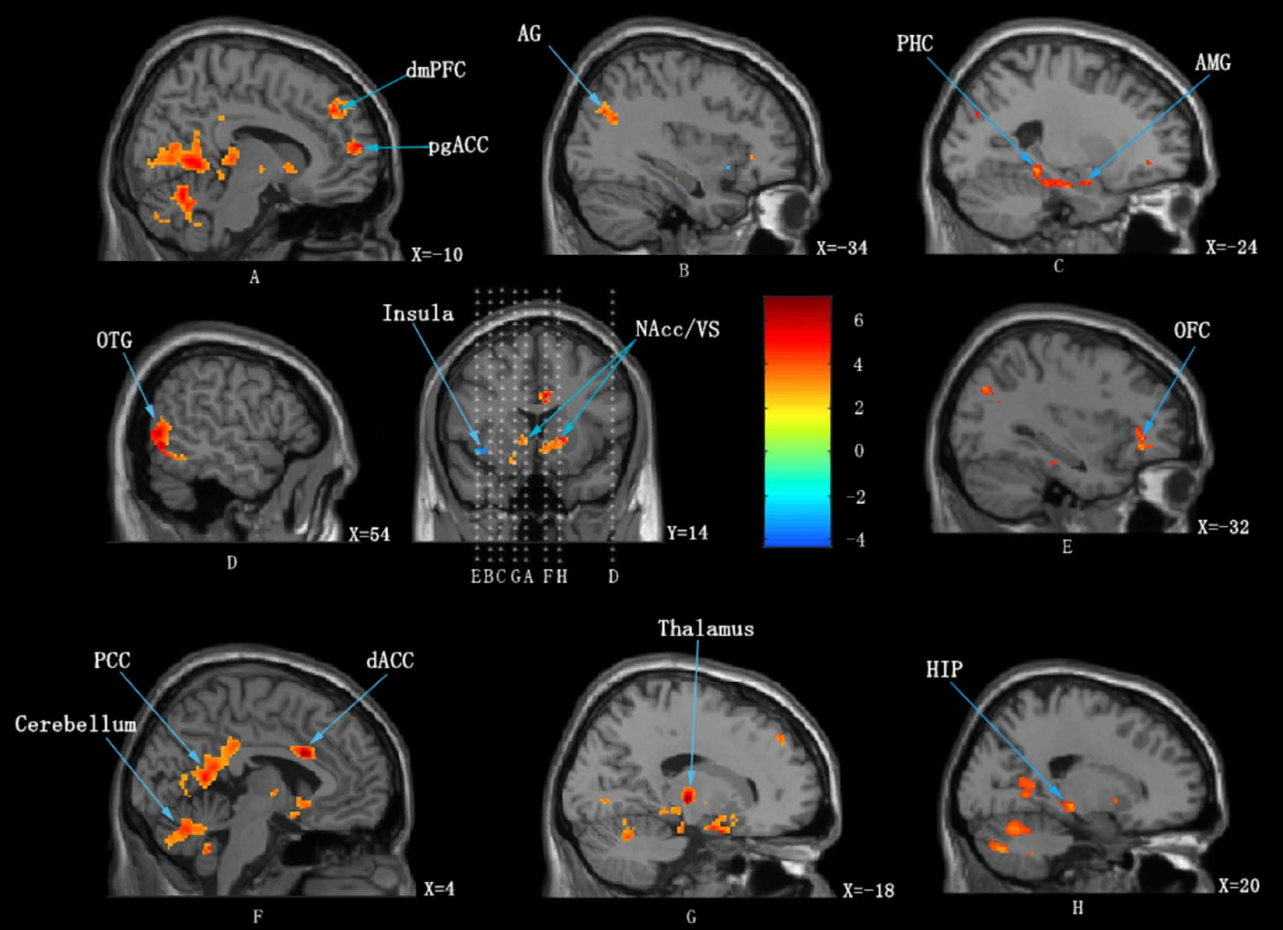
Increased (red) and decreased (blue) BOLD signal on viewing images of alcoholic beverages compared to viewing images of non-alcoholic beverages. dmPFC = dorsal medial prefrontal cortex; pgACC = pregenual anterior cingulate cortex; AG = angular gyrus; PHC = parahippocampus; AMG = amygdala; OTG = occipitotemporal gyrus; NAcc/VS = nucleus accumbens/ ventral striatum; OFC = orbital frontal cortex; PCC = posterior cingulate cortex; dACC = dorsal anterior cingulate cortex; HIP = hippocampus.

We observed that patients exhibited increased BOLD signal in response to alcoholic beverage cues compared to non-alcoholic beverage cues in the bilateral posterior cingulate cortex (PCC), right angular gyrus, left pregenual (pgACC) and right dorsal anterior cingulate cortex (dACC), left dorsomedial prefrontal cortex (dmPFC), left orbitofrontal cortex (OFC), right occipitotemporal gyrus (OTG), left angular gyrus (AG), right hippocampus (HIP), left parahippocampal gyrus (PHC), left amygdala (AMG), left nucleus accumbens (NAcc), right ventral striatum (VS), left thalamus and bilateral cerebellum. The left anterior insula (AIC) showed decreased BOLD signal in response to alcoholic beverage cues compared to non-alcoholic beverage cues. The results are displayed in Fig. 1 and the corresponding *p*-values are displayed in Table 2.

**Table 2:** Regions of activation in which alcohol related cues elicited greater activation than non-alcohol related cues (means *p* < 0.001 uncorrected, Cluster size >60 voxels).

Regions	*left/right*	*t*-score	Peak-level coordinates			Cluster size
			x	y	z	
Posterior Cingulate Cortex	L&R	10.62	−6	−58	−2	4142
Cerebellum	L&R	8.07	−4	−62	−16	
Dorsal Medial Prefrontal Cortex	L	4.75	−12	42	38	175
pregenual Anterior Cingulate Cortex	L	5.26	−10	60	14	115
Orbitofrontal Cortex	L	5.03	−32	34	−8	82
OccipitalTemporal Gyrus	R	6.66	54	−64	−4	488
Thalamus	L	6.62	−18	−18	2	88
Angular Gyrus	L	4.66	−34	7-70	32	186
Hippocampus	R	5.45	20	−24	−6	162
Parahippocampus,	L	5.31	−24	−36	−12	363
Amygdala	L	4.81	−20	−2	−20	
Dorsal Anterior Cingulate Cortex	R	6.74	4	14	28	117
Nucleus Accumbens	L	5.12	−4	0	−6	378
Ventral Striatum	R	5.01	16	14	0	
Insula	L	−4.22*	−40	2	−6	66

Coordinates are reported in Montreal Neurological Institute (MNI) coordinate.

*Means p = 0.001.

**Figure 2:**
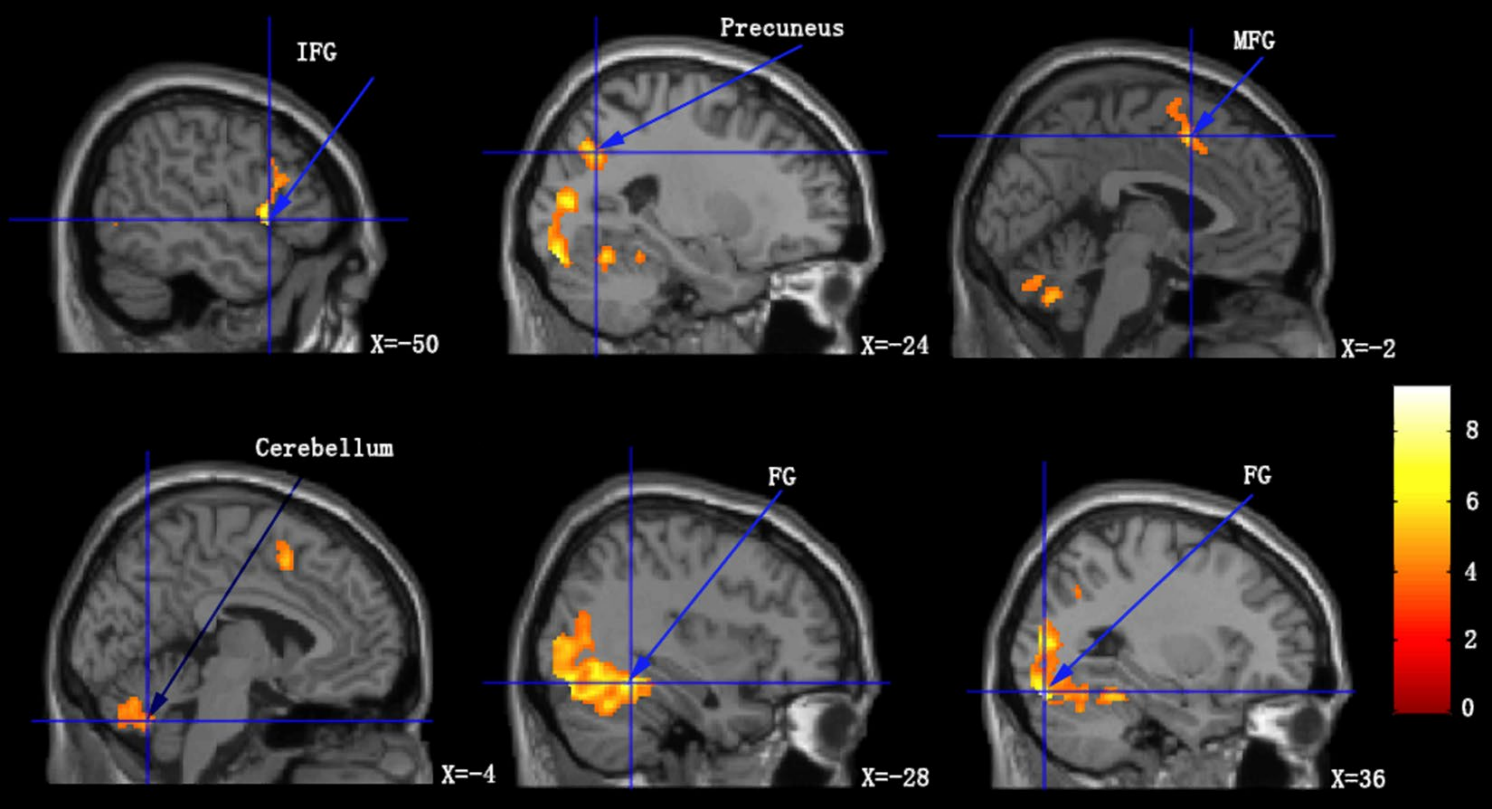
Increased BOLD signal on viewing images of alcoholic beverages and non-alcoholic beverages compared to viewing control images. MPG = medial fontal gyrus; FG = fusiform gyrus; IFG = inferior frontal gyrus.

Applying a conjunction analysis, we observed that patients exhibited increased BOLD signal in response to both alcoholic and non-alcoholic beverage cues in the left and right fusiform gyrus (FG), left inferior frontal gyrus (IFG), left medial frontal gyrus (MFG), left precuneus, and left cerebellum posterior lobe (Table 3; Fig. 2).

**Table 3:** Regions of activation in which both alcohol related cues and non-alcoholic related cues elicited (means *p* < 0.001 uncorrected, Cluster size >50 voxels).

Regions	*left/right*	*t*-score	Peak-level coordinates			Cluster size
			X	Y	Z	
Fusiform Gyrus	L	9.24	−28	−82	−18	2112
Fusiform Gyrus	R	7.82	36	−48	−16	2558
Inferior Frontal Gyrus	L	7.51	−50	14	0	510
Medial Frontal Gyrus	L	5.84	−2	12	50	147
Precuneus	L	5.83	−24	−66	36	139
Cerebellum Posterior Lobe	L	5.29	−4	−64	−36	246

Coordinates are reported in Montreal Neurological Institute (MNI) coordinate.

### Cue-reactivity fMRI functional connectivity

**Figure 3:**
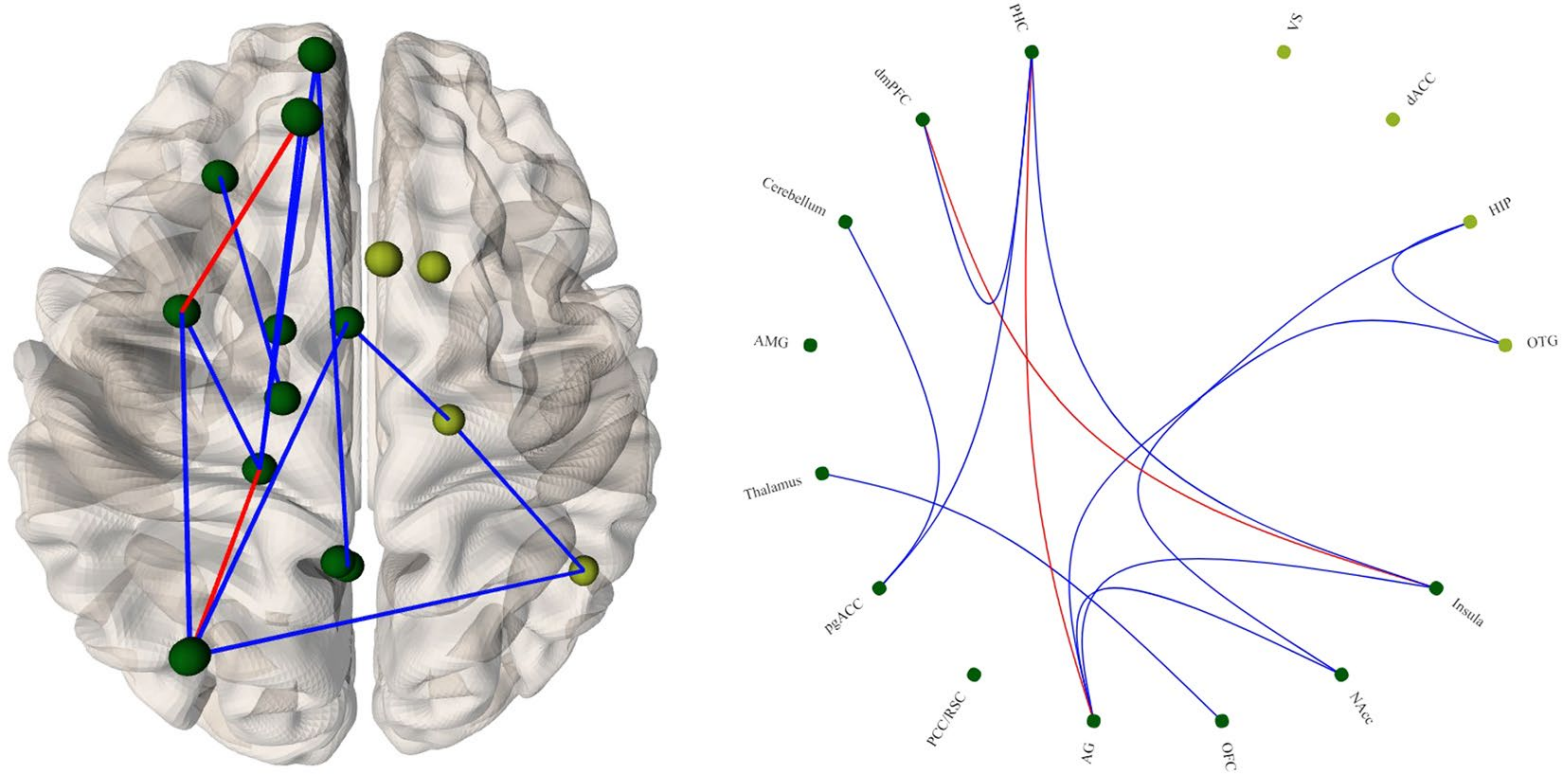
Changes in functional connectivity strength between on viewing alcoholic beverage images compared to viewing non-alcoholic beverage images based on cue-reactivity fMRI. Regions of interest on the right are shown in light green; regions of interest on the left are shown in dark green. Significantly increased connectivity is depicted in red and significantly decreased connectivity is depicted in blue.

Comparing the functional connectivity matrices in the alcoholic beverage and non-alcoholic beverage conditions, we observed increased functional connectivity between left dmPFC–left insula and left PHC–left AG. We further observed decreased functional connectivity in a network including left PHC, dmPFC, cerebellum, thalamus, pgACC, AG, OFC, NAcc, insula, right OTG, and the hippocampus (Fig. 3).

### Resting-state EEG activity

**Figure 4:**
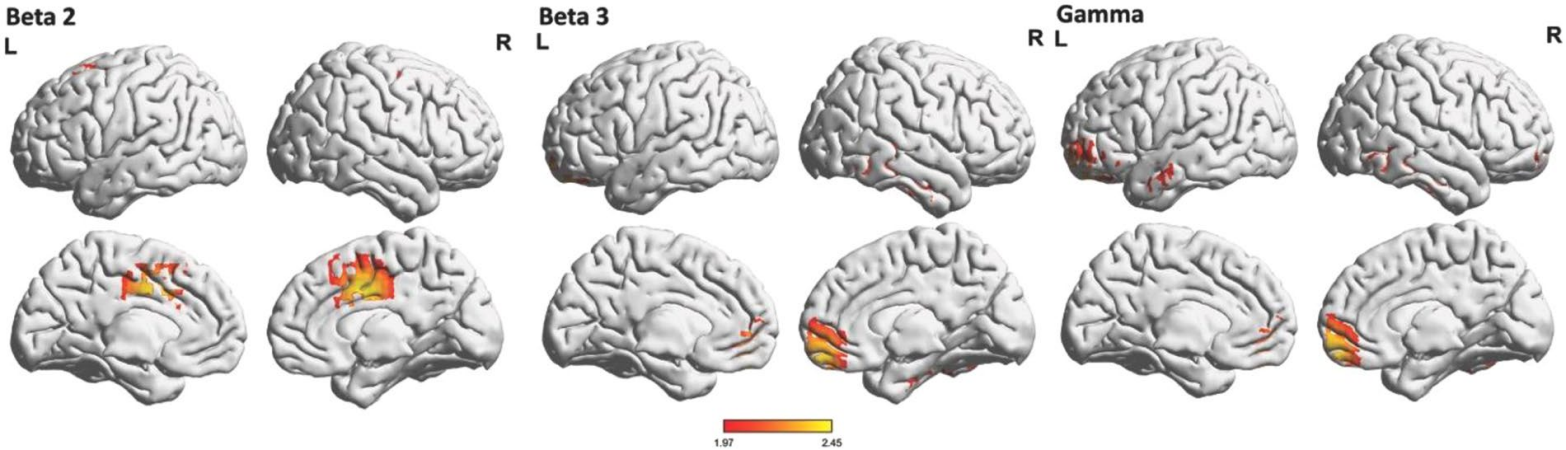
Increased resting-state EEG activity (red) for alcohol addicted patients compared to a normative databased in beta2 (18.5–21 Hz), beta3 (21.5–30 Hz) and gamma (30.5–44 Hz) frequency bands.

The resting-state EEG data showed significantly (*t* = 1.97, *p* < 0.05) increased beta2 activity in the left and right dACC, increased beta3 activity in the right pgACC extending to the ventromedial prefrontal cortex (vmPFC), and increased gamma activity in the right pgACC extending to the vmPFC, the left OFC, and frontal pole for patients with alcohol addiction in comparison to healthy controls (Fig. 4). No significant effects were obtained for the delta, theta, alpha1, alpha2, or beta1 frequency bands.

### Resting-state EEG functional connectivity

**Figure 5:**
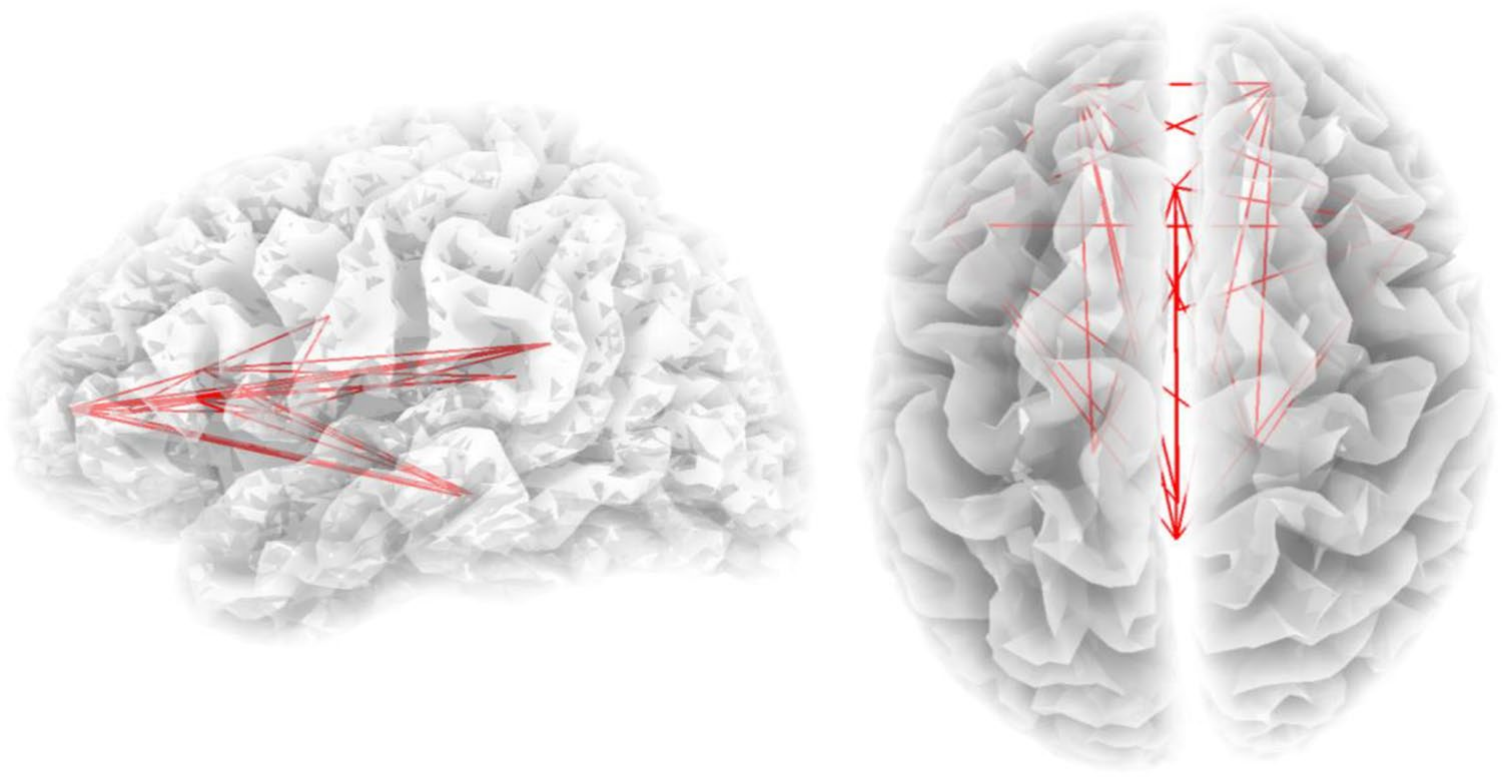
Increased functional connectivity in the theta (4–7.5 Hz) frequency band for alcohol patients compared to a normative database. Increased functional connectivity is depicted by the red lines.

We showed increased connectivity in the theta frequency band (t = 2.88, p < 0.05) (Fig. 5) for a densely-connected network consisting of the right OFC, pgACC, dACC, PCC, bilateral inferior frontal gyri (IFG), the left and right insula, and left PHC for patients with alcohol addiction in comparison to healthy controls. No significant effects were obtained for the delta, alpha1, alpha2, beta1, beta2, beta3, or gamma frequency bands.

## Discussion

The current study investigates the neural response to alcohol-related cues and resting electrical activity and functional connectivity among patients with alcohol addiction. The results of our study are consistent both across the two imaging modalities and with previously published results on resting-state and alcohol cue-reactivity paradigms. We observe increased BOLD signal levels associated with heightened cue-reactivity as well as corresponding cue-based functional dysconnectivity and resting state hyperactivity in specific cortical and subcortical regions demonstrating the response of the brain to the presence and absence of alcohol-related cues after a short period of abstinence in alcohol addicts. We further show hyperconnectivity in resting-state EEG functional connectivity and dysconnectivity in cue-based fMRI functional connectivity among a densely connected network of different alcohol-related brain areas supporting our hypothesis for a central craving network.

Alcohol dependence is an example of a learned response where the initial hedonic weight associated with alcohol is decreased due to excessive consumption of the substance^22^. This is proposed to result in a change in internal reference state from a normal to an addicted state, characterized by increased goal-directed attention or incentive salience towards its consumption^11,13^. The increased BOLD signal in the amygdala, PHC, NAcc and striatum on viewing pictures of alcoholic beverages as opposed to non-alcoholic beverages in the present study may be characteristic of the associative learning of substance abuse and the paradoxical reward associated with it^23^. The heightened PCC activity in the current study may be characteristic of a shift in internal state, i.e. allostasis. PCC is an important node in the default mode network and is shown to encode the allostatic shift to an addicted state in other substance addiction disorders^11,13^. The incentive salience to an otherwise neutral stimulus is hypothesized to be the component that drives the pathological craving of the substance. This is proposed to be modulated subcortically by the NAcc, which receives dopaminergic input from the VTA, which is strongly tied to reward-motivated behavior^24^. Thus, increased NAcc BOLD signal in the current study may be also be characteristic of the patients experiencing a pathological feeling of “want” upon seeing pictures of alcoholic beverages as opposed to non-alcoholic beverages^8^.

These subcortical regions also extend their dopaminergic input to more cortical regions such as dACC. dACC has been shown to encode goal-directed salience to an incoming stimulus, reward-based decision making, and learning and performance of novel tasks, which are functionally known to be dopaminergic^25^. Additionally a previous meta-analysis investigating the neural correlates of craving showed that dACC was an important area for encoding craving in different substance use disorders^26^. The pathological increase in BOLD signal and electrical activity in the dACC signifies its importance in encoding pathological cravings not only in response to a stimulus but also in a resting state. This may be confirmed by the results of our previous study where we showed that repetitive Transcranial Magnetic Stimulation (rTMS) targeting the dACC using a double cone coil or an electrode implanted on the dACC can dramatically suppress the intractable craving for alcohol^7,27^.

The emotional component of incentive salience is evaluated and modulated by pgACC and OFC. pgACC is shown to be involved in a top-down frontostriatal gating system that prevents erroneous or irrelevant sensory stimuli from reaching the cortex^28^. Dysfunction in this system has been shown to lead to various disorders such as tinnitus, chronic pain, etc.^29^. The pgACC has also been implicated to reduce aggression by exercising control over the amygdala and thus acts as a disorder-general suppression system^30^. pgACC and OFC are also proposed to evaluate the pleasure of salient stimuli and to prioritize behaviorally relevant stimuli^31^. The OFC itself has been shown to be involved in the assignment of reward and reward-based decision making along with the NAcc, VS, VTA, and the amygdala^32,33^. Thus, the pathological increase in both BOLD signal and beta activity in pgACC may be characteristic of dysfunctional suppression of craving. This may be confirmed with the results of our previous studies, which reported a reduction in this abnormal pgACC activity following rTMS targeting the dACC, leading to suppression of craving^7^. Additionally, hyperactivation in pgACC among heavy drinking college students may underlie greater attention and motivation for alcohol^34^, indicating that this region can predict relapse and thus is strongly related to craving^7,35,36^. The increases in BOLD signal and gamma activity in OFC may be characteristic of the paradoxical reward associated with salience and the integration of information from subcortical limbic regions to make reward-based decisions^32,37^.

An alternative explanation can be extrapolated from a recent study that looked at the neural circuits involved in flexible behavioral adaptations^38^. This study demonstrated that the reliability of the current behavioral strategy (actor) was encoded by the pgACC/vmPFC and the reliability of alternative strategies was encoded in parallel by the dorsolateral PFC^38^. If the reliability of an alternative behavioral strategy becomes greater than that of the actor, then the actor will be rejected by ventrolateral PFC. Subsequently, the dACC will switch to the new behavioral strategy and acceptance of this new behavioral strategy as the actor is mediated by NAcc^38^. Thus, both a medial and lateral track exist related to behavioral flexibility. The medial track consists of the vmPFC, pgACC, dACC, and VS (including NAcc). This track makes inferences about the actor that, through reinforcement learning, selects and learns the actions maximizing reward^38^. The lateral track consists of frontopolar cortex (FPC) and middle lateral PFC (mid-LPC). This track makes inferences about alternative strategies (~2–3) retrieved from long-term memory. The FPC infers the absolute reliability of these alternative strategies from action outcomes while the mid-LPC detects when one of them becomes reliable enough to be the new actor. Translating this to alcohol addiction, dACC switches to an addicted state (via habenula) and NAcc confirms this state as the new reference. The pgACC confirms the addicted state as reliable, and NAcc–pgACC functional connectivity maintains the addicted state. As a consequence of the addicted state being the reference, constant craving for alcohol will ensue in an attempt to maintain the addicted state as the reference.

The decrease in BOLD signal in the AIC is one that is different from previously published results in alcohol addiction. The insula is also a hub of the salience network along with the dACC^39^. Consistent with the increase in BOLD signal in the dACC, previous literature posits a concurrent increase in BOLD signal in the insula^40,41^. However, the insula is also a central controller of the autonomic nervous system^42^ and thus the observed decrease in BOLD signal in the insula could be a biomarker for compromised autonomic system activity in alcoholic patients. This is only a hypothesis, however, as we do not have any measures of heart rate variability or other autonomic system variables that can support this claim.

In addition to understanding the differences in BOLD signal in response to viewing alcoholic and non-alcoholic beverages, we also studied the similarities in viewing the two sets of visual cues by performing a conjunction analysis. We observed an increase in BOLD signal in the FG, IFG, MFG, precuneus and the cerebellum that was common to viewing pictures of alcoholic and non-alcoholic beverages. Although the FG is mainly recognized as a human face recognition area, previous research indicated has that when perceptual experts (such as car experts or bird experts) view the pictures of relevant stimuli (i.e. cars or birds), there is an increase in activity in the FG^43^. This indicates that the FG is an area that can process visual cues that a person is conditioned towards. The IFG and MFG are areas responsible for semantic processing^44^. This could be associated with patients reading the names of the beverages inside the scanner. The cerebellum is involved in motor control, hand-eye coordination, and higher-order cognitive functions^45^. Impairment of cerebellar function has been shown in substance abuse and other disorders such as schizophrenia, characterizing changes in personality traits and poor executive and working memory in patients^45,46^. Increased BOLD signal in the cerebellum may not be associated with visual processing but could just reflect impaired cerebellar function in alcoholics.

Further, the co-occurrence of beta-band EEG and BOLD signal in pgACC, vmPFC, and dACC not only indicates that these cortical areas are critical for craving but also indicates a potential relationship between beta-band activity and BOLD signal. Previous research has demonstrated that EEG beta activity is related to emotional and cognitive processes^47^, which reflects on the craving aspect of alcoholism. Also, in an fMRI and MEG study, researchers found convergent BOLD and beta-band activity in the fronto-limbic circuitry that underpins emotional cognitive processes^48^. Based on these previous findings, our result of convergent BOLD and beta-band activity in pgACC and dACC can strongly support that these cortical areas have an important role in processing emotional and cognitive aspect in alcoholism, i.e. craving.

Along with changes in BOLD signal and resting state EEG activity, we also observe significant changes in functional connectivity in both cue-reactivity and resting-state paradigms. In the cue-reactivity paradigm, we observe a disconnection between frontostriatal regions and regions of the executive control network. The pgACC/vmPFC is involved in top-down emotional regulation towards a stimulus and in this, case the pathological craving of alcohol^28^. The decreased connectivity between the frontostriatal regions suggests impaired regulation of pathological cravings as shown in another task in patients with alcohol dependency^49,50^. Furthermore, decreased resting-state functional connectivity in the executive control network has been shown as a correlate of decreased control over drinking and relapse in alcoholic patients^51,52^. The consistency of these results in cue-based functional connectivity also suggests an increase in pathological craving not only in resting state but also while exposed to a stimulus. This idea is confirmed by the increase in functional connectivity between similar cortical regions in the theta frequency band. Theta has been consistently associated with substance-related disorders, especially during withdrawal and craving states, as well as in several basic motivational processes^53,54^. In addition, substance abusers have been shown to exhibit bilateral hypersynchronization in the theta frequency band in the frontoposterior and frontotemporal regions compared to healthy adults^55,56^. Further, theta has been associated with long-distance transmission of information, integrating it from several regions^57^. Consistent with these results, the increased functional connectivity in the theta frequency band between regions associated with craving in the current study bolsters the hypothesis of a central craving network, where different regions encoding different aspects of alcohol addiction such as the emotional “want”, “reward”, and “pleasure” as well as neurobiological “incentive salience” are possibly maladaptively integrated into a unified percept by long-distance theta connectivity. It is important to note here that previous research shows an inverse relationship between BOLD signal activity and the lower frequencies of ongoing EEG activity^58^. In the current study, we observe that this inverse relationship holds even for functional connectivity. It is also important to keep in mind that here we compare resting-state EEG and task-based fMRI and so further research is required to confirm this inverse relationship between EEG and fMRI functional connectivity.

One of the main advantages of this study is the combination of techniques which each have superior spatial and temporal resolution, respectively. Thus, we investigate pathological craving after 24 hours of alcohol abstinence from two different angles. The first is to understand the changes in spontaneous activity and functional connectivity. The second is to understand the changes in activity and functional connectivity in response to alcoholic and non-alcoholic cues. The superior spatial resolution of fMRI helps us investigate deeper structures and their relevance to pathological craving in alcohol dependence on exposure to alcohol-related cues. Conversely, the superior temporal resolution of EEG helps us to understand the changes in different frequency bands. The combination of these two techniques in the same patients gives us a more comprehensive view of pathological craving and the functional networks that may encode it. Thus, from the results of the current study we observe that different cortical and subcortical regions are involved in encoding different aspects of substance craving. The incentive salience encoded by the dACC is characteristic of “give me more”, which is not suppressed by the dysfunctional pgACC suppression mechanism^59,60^. The result is the positive reinforcement of a feeling of arousal and reward towards alcohol encoded by the limbic structures and OFC.

However, the study also has some limitations. For instance, our study features a small sample size and lacks a control group for the cue-reactivity paradigm. Future studies focusing on these two primary limitations and confirming the results with resting state fMRI functional connectivity and task-based EEG may be able to confirm the presence of a craving network. These results also pave the way for other network-based approaches such as correlation analysis, directed functional connectivity, dynamic functional connectivity, etc. to confirm the correlates of pathological craving. Nevertheless, the current study is one of the first to combine resting-state EEG and cue-based fMRI to present the neural correlates of substance craving with superior spatial and temporal resolution and propose a substance-general craving network.

## Conclusion

Our results reveal significant changes in both cue-based fMRI reactivity, resting-state neural activity, and functional connectivity which represent the neural correlates of pathological craving in alcohol addiction. According to previous literature, alcohol addiction is a learned response wherein the hedonic weight to stimuli is decreased on excessive consumption of the substance by associating it with a paradoxical reward. This results in a shift in the normal, healthy self-referential state to an addicted state characterized by an incentive salience, which behaviorally manifests as a pathological craving for the substance. This craving is strongly associated with an emotional component and thus results in relapse of excessive alcohol consumption following even a short period of abstinence. In the current study, we propose that these different aspects of substance-related craving are encoded by a central craving network characterized by changes in activity and functional connectivity of the NAcc, VS, amygdala, PHC, PCC, dACC, pgACC, and OFC. The associative learning of the paradoxical reward may be mediated by the regions of the limbic and medial temporal lobes; the shift in self-referential state may be encoded by the PCC; the incentive salience may be encoded by limbic regions and dACC; the emotional component may be encoded by the limbic regions along with the pgACC and OFC. Thus, craving may be hypothesized as the unified percept of incentive salience related to reward, lack of suppression of this salience, and the emotional association with reward.

## Materials and Methods

### Patient demographics

Eleven patients (N = 11; 8 males and 3 females) with a mean age of 48.12 years (sd = 8.54) were included in the study. The mean score on the NRS (numeric rating scale) for the desire of alcohol was 8.32 (sd = 1.87) and the mean Audit (Alcohol Use Disorders Identification test) score was 36.21 (sd = 5.89). An Audit (Alcohol Use Disorders Identification test) score of 20 or above clinically warrants further diagnostic evaluation for alcohol dependence. All patients were first screened by a psychiatric consultation. Following that an EEG was performed, as well as an fMRI using images of alcoholic and non-alcoholic beverages as cues. All patients reported that they had undergone multiple treatments with only temporary success, due to major craving and an obsessive-compulsive focus on alcohol, presented with a relapsing clinical picture. All patients were resistant to acamprosate and naltrexone. Patients reported that during relapses they engaged in binge drinking associated with aggression with amnesia, mood and sleep problems followed by withdrawal symptoms upon arresting alcohol intake. Behavioral treatment in outpatient individualized sessions, group sessions, and inpatient treatment in a general hospital as well as a specialized addiction hospital all yielded only temporary success. See Table 1 for Table demographics, anthropometric and laboratory measures.

**Table 1:** Demographics, anthropometric and laboratory measures for alcohol addicted participants.

	**Alcohol addicted**
	(n = 11)
Age (years)	48.12 (8.54)
Gender	♂ 8 ♀ 3
Body weight (kg)	73.30 (10.55)
Height (cm)	175.21 (12.38)
BMI	23.9 (2.05)
NRS Desire alcohol^1^	8.32 (1.87)
Audit^2^	36.21 (5.89)
Cholesterol (mmol/L)	4.77 (0.85)
Triglycerides (mmol/L)	1.65 (1.19)
HDL (mmol/L)	1.47 (0.43)
GGT (U/L)	91.79 (72.63)
ALT (U/L)	43.07 (30.39)
AST (U/L)	39.14 (34.28)
Glucose (mmol/L)	5.04 (0.54)

^1^Numeric rating scale (how much do you desire for alcohol?).

^2^Audit (Alcohol Use Disorders Identification test) scores of 20 or above clearly warrant further diagnostic evaluation for alcohol dependence.

Participants were requested to refrain from alcohol consumption 24 hours prior to recording and from caffeinated beverage consumption on the day of recording. This study was approved by the local ethical committee (Antwerp University Hospital) and was in accordance with the declaration of Helsinki. Written informed consent was obtained from all patients.

### Functional Magnetic Resonance Imaging

#### Data Acquisition

The patients underwent three fMRI sessions at different time points on a 3 T MRI scanner (ACHIEVA, Philips Medical Systems, Best, The Netherlands). Alcohol addicted patients performed a visual cue stimulation experiment using a blocked designed paradigm. During the experiment, the fMRI was performed to map out BOLD signals in different cortical areas. Each patient had four sessions and each session contained 120 scans (EPI; 120 dynamic scans, repetition time (TR) = 3000 ms; echo time (TE) = 33 ms; voxel size = 2.9 × 2.9 × 4.0 mm; field of view (FOV) = 230 × 230 mm; flip angle (FA) = 90). Within and between sessions balance was included in the experimental design. In each session, 120 images were divided into 24 blocks and 4 conditions. Each block contained 5 images within the same condition and each image lasted 3 s. The four conditions were (a) alcohol condition (images of alcohol beverages), (b) beverage condition (images of non-alcoholic beverages), (c) control condition (unidentifiably scrambled images of alcoholic and non-alcoholic beverages), and (d) fix condition (image of a fixation cross). Patients were instructed to focus on the visually presented stimuli throughout the entire experiment in the MRI machine. Numerous studies have used a cue-reactivity paradigm, which exposes subjects to drug-related stimuli, while monitoring their physiological responses to investigate addictive behavior. A meta-analysis of the cue-reactivity paradigm suggests it can generate a stable profile of significant addict-related effects^61^. To improve our understanding of alcohol addictive behavior, establish treatment for active alcohol users, and prevent relapse, it is important to understand the neurobiological mechanism that underpins alcohol-related behavior.

#### Data analysis

The fMRI data were analyzed using Statistical Parametric Mapping 8 (SPM8) (The Wellcome Trust Center for Neuroimaging, London, UK) (http://www.fil.ion.ucl.ac.uk/spm). The structural T1 image (retaining the gray matter, white matter, and CSF) was first skull-stripped. The functional images were realigned to the first scan to correct for head movement. The high-resolution skull-stripped T1 image was then co-registered to the mean realigned functional image and subsequently normalized to the Montreal Neurological Institute (MNI) coordinates. Finally, spatial smoothing was applied using a 6 × 6 × 6 mm full-width at half maximum (FWHM) of the Gaussian smoothing kernel. The functional activation was analyzed using a standard general linear model (GLM). A boxcar reference function was convolved with the hemodynamic function to represent the time course of the expected signal during the experiment.

The first blood oxygenation level-dependent (BOLD) signal was generated by a one-sample t-test specified to a contrast between seeing images of alcoholic beverages versus images of non-alcoholic beverages in the patient group. The second blood oxygenation level-dependent (BOLD) signal was generated by a conjunction analysis assessing a comparison of viewing images of alcoholic beverages to control images versus a comparison of viewing images of non-alcoholic beverages to control images across all patients. The one-sample t-test and conjunction analysis were both conducted at voxel level and a threshold for significant functional activation at *p* < 0.01 uncorrected for the one-sample t-test and *p* < 0.001 uncorrected for the conjunction analysis. The set of cluster size was larger than 60 voxels in the one-sample t-test and larger than 50 voxels in the conjunction analysis.

### Functional connectivity

The average beta values for every condition at the peak activation voxel of all regions of interest were extracted from each individual and activity of all regions of interest were correlated with one another to create a co-activation matrix. This co-activation matrix was the measure of functional connectivity in the cue-based fMRI paradigm. The functional connectivity matrix in the alcoholic beverage and non-alcoholic beverage conditions were compared using Fischer’s Z and *p* < 0.05.

### Electroencephalography

#### EEG Data collection

EEG data were obtained as a standard procedure. Recordings were obtained in a fully lighted room with each participant sitting upright on a small but comfortable chair. The actual recording lasted approximately five minutes. The EEG was sampled using Mitsar-201 amplifiers (NovaTech http://www.novatecheeg.com/) with 19 electrodes placed according to the standard 10–20 International placement (Fp1, Fp2, F7, F3, Fz, F4, F8, T7, C3, Cz, C4, T8, P7, P3, Pz, P4, P8, O1, O2). Participants abstained from alcohol consumption 24 hours prior to EEG recording and from caffeinated beverages on the day of recording to avoid alcohol-induced changes in EEG^62^ or a caffeine-induced alpha power decrease^63,64^. The vigilance of participants was monitored by EEG parameters such as the slowing of alpha rhythm or the appearance of spindles as drowsiness that is reflected in enhanced theta power^65^. Impedances were checked to remain below 5 kΩ. Data was collected eyes-closed (sampling rate = 500 Hz, band passed 0.15–200 Hz). Off-line data were resampled to 128 Hz, band-pass filtered in the range 2–44 Hz and subsequently transposed into Eureka! software^66^, plotted and carefully inspected for manual artifact-rejection. All episodic artifacts including eye blinks, eye movements, teeth clenching, body movement, and other ECG artifacts were removed from the stream of the EEG. In addition, an independent component analysis (ICA) was conducted to further verify if all artifacts had been excluded. To investigate the effect of possible ICA component rejection, we compared the power spectra with two approaches: (1) after visual artifact rejection only and (2) after additional ICA component rejection. The mean power in the delta (2–3.5 Hz), theta (4–7.5 Hz), alpha1 (8–10 Hz), alpha2 (10–12 Hz), beta1 (13–18 Hz), beta2 (18.5–21 Hz), beta3 (21.5–30 Hz), and gamma (30.5–44 Hz) bands^67–69^ did not show a statistically significant difference between the two approaches. Average Fourier cross-spectral matrices were computed for all eight bands.

#### Normative database

A normative database was used. Exclusion criteria were known psychiatric or neurological illness, psychiatric history of drug/alcohol abuse in a participant or any relative, current psychotropic/CNS active medications, history of head injury (with loss of consciousness) or seizures, headache, and physical disability. About 3–5 min of EEG was continuously recorded while participant sat with their eyes closed on a comfortable chair in a quiet and dimly lit room. EEG data were acquired at the 19 standard leads prescribed by the 10–20 international system (FP1, FP2, F7, F3, FZ, F4, F8, T3, C3, CZ, C4, T4, T5, P3, PZ, P4, T6, O1, O2) using both earlobes as reference and enabling a 60 Hz notch filter to suppress power line contamination. The resistance of all electrodes was kept below 5 kΩ. Data of the BRL database were acquired using the 12-bit A/D BSA acquisition system (Neurometrics, Inc., New York, NY) and sampled at 100 Hz. For consistency, we subsequently up-sampled the BRL database to 128 Hz using a natural cubic spline interpolation routine (Congedo *et al*., 2002). We removed from all data biological, instrumental and environmental artifacts, paying attention to biological artifacts generated by the eyes, the heart and the muscles of the neck, face and jaw. EEG recordings were visually inspected on a high-resolution screen and epochs containing visible artifacts were marked and ignored for ensuing analysis.

#### Source localization

Standardized low-resolution brain electromagnetic tomography (sLORETA^70,71^) was used to estimate the intracerebral electrical sources. As a standard procedure, a common average reference transformation^70^ was performed before applying the sLORETA algorithm. sLORETA computes electric neuronal activity as current density (A/m2) without assuming a predefined number of active sources. The solution space used in this study and associated lead-field matrix are those implemented in the LORETA-Key software (freely available at http://www.uzh.ch/keyinst/loreta.htm). This software implements revisited realistic electrode coordinates (Jurcak *et al*. 2007) and the lead field produced by Fuchs *et al*.^72^ by applying the boundary element method on the MNI-152 (Montreal neurological institute, Canada) template of Mazziotta *et al*.^73,74^. The sLORETA-key anatomical template divides and labels the neocortical (including hippocampus and anterior cingulate cortex) MNI-152 volume in 6,239 voxels of dimension 5 mm^3^, based on probabilities returned by the Demon Atlas^75,76^.

#### Lagged phase coherence

Coherence and phase synchronization between time series corresponding to different spatial locations are usually interpreted as indicators of “connectivity”. However, any measure of dependence is highly contaminated with an instantaneous, non-physiological contribution due to volume conduction^77^. However, Pascual-Marqui^78^, introduced new measures of coherence and phase synchronization taking into account only non-instantaneous (lagged) connectivity, effectively removing the confounding factor of volume conduction. Such “lagged phase coherence” between two sources can be interpreted as the amount of cross-talk between the regions contributing to the source activity^79^. Since the two components oscillate coherently with a phase lag, the cross-talk can be interpreted as information sharing by axonal transmission. More precisely, the discrete Fourier transform decomposes the signal in a finite series of cosine and sine waves at the Fourier frequencies (Bloomfield 2000). The lag of the cosine waves with respect to their sine counterparts is inversely proportional to their frequency and amounts to a quarter of the period; for example, the period of a sinusoidal wave at 10 Hz is 100 ms. The sine is shifted a quarter of a cycle (25 ms) with the respect to the cosine. Then the lagged phase coherence at 10 Hz indicates coherent oscillations with a 25 ms delay, while at 20 Hz the delay is 12.5 ms, etc. The threshold of significance for a given lagged phase coherence value according to asymptotic results can be found as described by Pascual-Marqui (2007), where the definition of lagged phase coherence can be found as well. As such, this measure of dependence can be applied to any number of brain areas jointly, i.e., distributed cortical networks, whose activity can be estimated with sLORETA. Measures of linear dependence (coherence) between the multivariate time series are defined. The measures are non-negative, and take the value zero only when there is independence and are defined in the frequency domain: delta (2–3.5 Hz), theta (4–7.5 Hz), alpha1 (8–10 Hz), alpha2 (10–12 Hz), beta1 (13–18 Hz), beta2 (18.5–21 Hz), beta3 (21.5–30 Hz) and gamma (30.5–44 Hz). Based on this principle, lagged linear connectivity was calculated. Time-series of current density were extracted for different regions of interests using sLORETA. Power in all 6,239 voxels was normalized to a power of 1 and log transformed at each time point. Region of interest values thus reflect the log-transformed fraction of total power across all voxels, separately for specific frequencies. Regions of interest selected were based on the activity findings of both EEG and fMRI - left and right orbitofrontal cortex, the pregenual anterior cingulate cortex, the dorsal anterior cingulate cortex, the posterior cingulate cortex, the left and right inferior frontal gyrus, the left and right insula, the left parahippocampus.

#### Statistical analyses

The methodology used is a non-parametric permutation test. It is based on estimating, via randomization, the empirical probability distribution for the max-statistic, under the null hypothesis comparisons^80^. This methodology corrects for multiple testing (i.e. for the collection of tests performed for all voxels, and for all frequency bands). Due to the non-parametric nature of this method, its validity does not rely on any assumption of Gaussianity^80^. The significance threshold for all tests was based on a permutation test with 5000 permutations. Comparisons were made between the healthy controls versus patients with an alcohol addiction. These comparisons were performed on a whole brain by sLORETA statistical contrast maps through multiple voxel-by-voxel comparisons in a logarithm of *t*-ratio.

### Data availability statement

The datasets generated during and/or analyzed during the current study are available from the corresponding author on reasonable request.
